# Differential Recruitment of Brain Networks following Route and Cartographic Map Learning of Spatial Environments

**DOI:** 10.1371/journal.pone.0044886

**Published:** 2012-09-18

**Authors:** Hui Zhang, Milagros Copara, Arne D. Ekstrom

**Affiliations:** 1 Center for Neuroscience, University of California Davis, Davis, California, United States of America; 2 Neuroscience Graduate Group, University of California Davis, Davis, California, United States of America; 3 Department of Psychology, University of California Davis, Davis, California, United States of America; University of Regensburg, Germany

## Abstract

An extensive neuroimaging literature has helped characterize the brain regions involved in navigating a spatial environment. Far less is known, however, about the brain networks involved when learning a spatial layout from a cartographic map. To compare the two means of acquiring a spatial representation, participants learned spatial environments either by directly navigating them or learning them from an aerial-view map. While undergoing functional magnetic resonance imaging (fMRI), participants then performed two different tasks to assess knowledge of the spatial environment: a scene and orientation dependent perceptual (SOP) pointing task and a judgment of relative direction (JRD) of landmarks pointing task. We found three brain regions showing significant effects of route vs. map learning during the two tasks. Parahippocampal and retrosplenial cortex showed greater activation following route compared to map learning during the JRD but not SOP task while inferior frontal gyrus showed greater activation following map compared to route learning during the SOP but not JRD task. We interpret our results to suggest that parahippocampal and retrosplenial cortex were involved in translating scene and orientation dependent coordinate information acquired during route learning to a landmark-referenced representation while inferior frontal gyrus played a role in converting primarily landmark-referenced coordinates acquired during map learning to a scene and orientation dependent coordinate system. Together, our results provide novel insight into the different brain networks underlying spatial representations formed during navigation vs. cartographic map learning and provide additional constraints on theoretical models of the neural basis of human spatial representation.

## Introduction

Humans can learn the spatial properties of the surrounding environment either by directly navigating it or by studying it from a cartographic map. During navigation, we typically determine the path to our goal based on remembering past trajectories and/or deriving novel paths to our intended goal, both of which we refer to here as “route learning.” During map learning, we can visualize the relations of objects in an environment from a single overview perspective, which we can then use to derive paths to our goal. We refer to this type of learning as “map learning” and is also commonly referred to as “survey learning” [1]. Both of these forms of learning can be thought of as contributing to a “cognitive map,” a representation of a spatial environment that is referenced primarily to landmarks or other external coordinates [2], [3], [4].

Previous behavioral studies provide support for the idea that participants improve differentially on spatial measures following direct navigation vs. map learning. For example, several studies showed that studying a spatial layout from a map improved estimation of Euclidean distances between remote objects compared to route learning [5], [6], [7], [8], [9], [10]. In contrast, learning the same layouts by directly navigating them improved estimation of distances of actual paths traversed compared to survey learning [10]. These findings have been interpreted to suggest that route learning favors a more trajectory-specific form of representation while survey learning favors more geometrically based, landmark-referenced representations. While some reports in the human spatial navigation behavioral literature thus support the idea that representations formed following route and survey learning involve different behavioral properties, whether and how the brain systems differ for cognitive maps derived from route vs. cartographic map learning remains unclear.

One influential model of how we represent spatial information postulates that spatial representations emerge via converging cognitive systems during route and cartographic map learning. According to this proposal, navigation initially involves representation of landmarks with routes and, with sufficient exposure, provides a configural map of the environment. Cartographic map learning involves similar means of representation [11], [12], or perhaps more immediate access to configurations of landmarks and routes within an environment [5], but provides the same eventual configural knowledge, the “survey representation.” One prediction of this model is that neural representations formed following sufficient route or map learning should not differ substantially. Consistent with this proposition, the few studies conducted on the neural basis of map representation using fMRI have often been interpreted to support this idea.

In one such study, Wolbers and Buchel (2005) had participants view videos of navigation through a virtual environment, point to the locations of stores using other stores as a reference, and then draw aerial maps of the environment following their fMRI session [1]. By the end of the session, subjects drew highly accurate maps of their environment. Furthermore, retrosplenial activation correlated with improvements in pointing to store locations across sessions, leading the authors to infer the importance of retrosplenial cortex in survey representation. In studies by Shelton and Gabrieili (2002) and Shelton and Pippit (2007), participants underwent fMRI while they viewed videos of navigation from a 1) fixed aerial-perspective 2) variable aerial perspective 3) route-based perspective and then performed a scene recognition task outside of the scanner in which they viewed images during a recognition task from the same and different experienced perspectives. Analyses compared activation during viewing of images and videos from route and survey-based perspectives that were subsequently successfully recognized during the scene recognition task. The authors reported that the survey condition activated the same brain areas as the route condition while the route condition activated a larger number of areas overall compared to the survey condition [13], [14]. The authors concluded that the brain areas involved in survey representation involved a subset of the brain areas recruited during navigation (see also: [15]). These results are often interpreted to support the idea that the neural systems involved in route and cartographic map learning typically share a high-degree of overlap.

An alternative to the above conceptualization of route and cartographic map learning is that spatial representations formed following route learning are typically more scene and trajectory dependent while those formed by studying cartographic maps are typically more geometrically anchored (e.g., [10]). This proposal regarding differences in route and survey learning is consistent with the notion that representations formed following cartographic map learning involve a more object-referenced form of memory because a collection of landmarks can be coded as a single geometrical shape [10], [16]. In contrast, those formed following route learning depend on memory for individual trajectories [5], [10]. Previous fMRI studies, however, did not test representations following route and cartographic map learning under conditions that would differentially tap into the their scene and geometrical dependence, respectively. Thus, it could be that the networks underlying route and map learning show more profound differences than previously demonstrated in prior neuroimaging work if tested under conditions tapping separately into scene and landmark-referenced memory, respectively.

To address this issue, we adapted two tests of spatial memory that have been used extensively in the human spatial navigation behavioral literature although significantly less frequently in the human spatial navigation neuroimaging literature. Specifically, we adapted two conditions from a paradigm used by Waller and Hodgson (2006) and others [17], [18], [19] to differentially measure scene and orientation dependent perceptual memory and landmark-referenced memory, respectively. In the Waller and Hodgson paradigm, participants first studied the positions of objects arranged as a spatial layout from the center of a room [20]. Participants were then blindfolded and tested in two different pointing tasks. In the orientation-dependent egocentric pointing task, participants pointed to the locations of objects in the room (“point to X”) from a location they chose; participants thus retrieved the spatial location of target objects based on being oriented in the room and their short-term memory for the scene from that perspective. In a second condition, involving judgments of relative direction (JRD), participants were told to imagine themselves at a specific object X, facing object Y, and to point to object Z. Participants thus retrieved the spatial relations by referencing between multiple different objects. Using their scene memory was difficult in this situation because they were disoriented from their previous position. Based on a double dissociation between the pointing tasks (egocentric vs. JRD) and conditions (oriented vs. disoriented), the authors concluded that humans possess two different forms of spatial representation: a transient perceptually-based system dependent on orientation and an enduring but courser long-term representation system dependent on representation of the relative positions of landmarks (see also: [17], [19], [21]).

**Figure 1:**
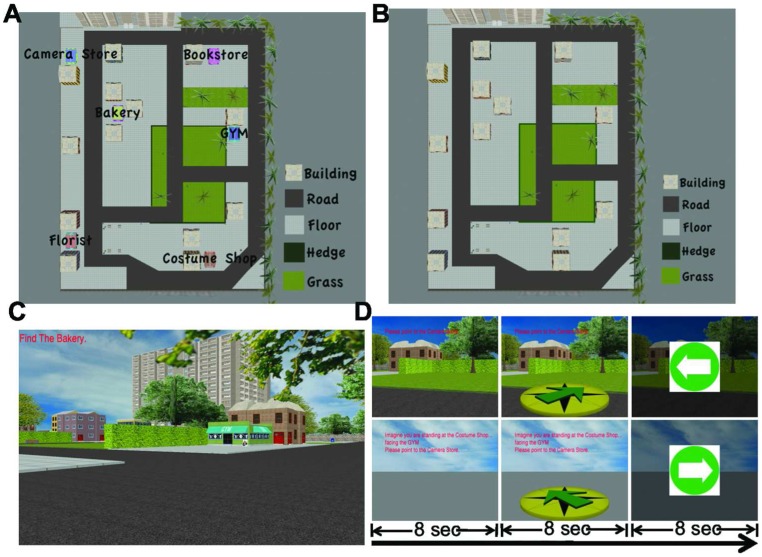
Materials and design. A) Map of one of the layouts used in our study. B) Target stores removed from the same layout in Figure 1A. C) Route view of the layout in Figure 1A. D) Set-up of the scene and orientation dependent perceptual (SOP) pointing task and judgment of relative direction (JRD) of landmarks pointing task. Example question during the SOP task: “Point to the Costume Party.” Example question during the JRD task: “Imagine you are standing at the Costume Shop, facing the Gym. Please point to the Camera Store.”

We adapted elements of the Waller and Hodgson paradigm to virtual reality to assess what insight they might provide into these two forms of representation following route vs. map learning. The first task was primarily dependent on being oriented in the environment based on the perceptual details of the scene, which we termed the “scene-dependent, orientation-dependent perceptual” (SOP) pointing task. The second task was dependent on knowledge of the relative position of landmarks to each other and not being oriented in the immediate environment with scene information, which we termed the judgment of relative direction (JRD) of landmarks pointing task. During map learning, participants in our study viewed the spatial layout from an aerial perspective, repeatedly drawing maps to ensure mastery of the spatial layout. During route learning, participants repeatedly drove through the virtual environment and were tested on their knowledge of the layout while driving (Figure 1; see Material and Methods).

Previous behavioral studies employing measures that primarily tapped into orientation-dependent and landmark-referenced memory showed differences in how route vs. map learning affected these two forms of representation. Taylor et al. (1999) had participants study maps or navigate an unfamiliar campus building for 10–20 minutes [10]. Participants that learned the environment by route navigation showed better estimates of path distances and worse estimates of Euclidean distances while the opposite pattern emerged for map learners, who showed better estimates of Euclidean distance and worse estimates of path distances (see also: [22]). Because path estimation requires orientation within the environment, while Euclidean distance estimation requires knowledge of direction and distances of landmarks, these studies point to a differential effect of route and map learning on orientation-dependent and landmark-referenced memory, respectively. Together, these findings suggest that scene-dependent perceptual memory improves differentially following route learning while landmark-referenced long-term memory improves differentially following map learning. These previous behavioral studies, though, did not look at the neural basis of these representations, an issue we address here.

To address whether brain networks differ during the SOP and JRD tasks following route and map learning, we first had subjects learn two different environments by extensive route and map learning, respectively (see Methods). This involved learning the environment by either navigating or studying a map. During neuroimaging, subjects then performed the SOP and JRD tasks on the two different environments. Since the critical manipulation we employed was whether subjects had learned one of the two environments through route or map learning, comparisons within SOP and JRD tasks were balanced in terms of visual input (during encoding, however, *prior* to imaging, the environments were learned from either a route or aerial perspective, and thus necessarily not matched in terms of visual rendering). This allowed us to investigate how learning in the two conditions directly affected retrieval separately within the two tasks. The SOP and JRD tasks, however, necessarily differed substantially in terms of visual input, as well as perceptual and memory demands. Thus, comparisons between SOP and JRD tasks, and subsequent activations derived from these contrasts, could have multiple determinants and thus we are cautious in any inferences based on directly comparing the two tasks. One specific prediction we test regarding route and map learning is that if route learning involves greater dependence on scene information than map learning, we expect differences in brain areas involved in scene representation following route compared to map learning (parahippocampal cortex, retrosplenial cortex). Specifically, we predicted that this difference between route and map learning would be most pronounced when subjects converted a scene-dependent representation acquired during route learning to a landmark-referenced representation during the JRD task. In contrast, we predicted that map learning, compared to route learning, would differentially recruit brain areas involved in converting from more geometrically anchored representations to those involved in scene-based representation. Specifically, we predicted differential activations during the SOP task following map compared to route learning.

## Results

### Behavioral Results

To gain insight into the accuracy of representations utilized during the SOP and JRD tasks following route and map learning, we compared mean pointing error in the SOP and JRD tasks. We employed configuration error in our analysis of the SOP task because it corrects for the fact that participant representations may be rotated relative to their actual pointing position although their representation is otherwise accurate (e.g., rotated 180**°** but still accurate, [17]). The overall trend of the results, however, was similar with absolute pointing error (Table 1). A 2×2 (learning method [route vs. map learning]×(pointing task [SOP vs. JRD]) repeated-measures ANOVA revealed a main effect of encoding, indicating that performance after route learning was better than that following map learning (*F(1,15)* = 5.1, *p* = 0.04, *MSE* = 88, Table 1), which was driven primarily by higher performance in the SOP task for route than map learning (*t(15)* = 2.1, p = 0.05). No other differences were significant (Table 1). Poorer pointing accuracy in the SOP task following map compared to route learning was anticipated because participants had not experienced any of the viewpoints previously following map learning but had direct experience with them following route learning. To account for differences in behavioral performance participant error rates were used as covariates in our fMRI-ANOVA analysis. This allowed us to look for patterns of activations in the brain while controlling for potential differences in performance. We note that overall participant pointing accuracy in both the SOP and JRD tasks was high, with mean pointing error typically about 25**°**, which was well above chance (chance performance = 90°). We did not find any differences in reaction time between the two tasks (Table 1).

**Table 1:** Mean (standard deviation) absolute pointing error for SOP and JRD tasks, mean configuration error, and mean response latency across subjects (note: we could not measure configuration error for the JRD task because there were no orienting stimuli from which to calculate this measure).

	Route learning		Map learning	
	SOP pointing error	JRD pointing error	SOP pointing error	JRD pointing error
Absolute pointing error (deg)	20.16 (16.98)	27.38 (17.53)	30.57 (31.63)	29.61 (16.75)
Configuration error (deg)	18.16 (6.32)	N/A	26.48 (16.62)	N/A
Response latency (second)	11.3 (0.6)	11.6 (0.5)	11.4 (0.6)	11.4 (0.8)

Note that response latency is measured from the beginning of the trial.

### Differences in Brain Activations Following Route and Map Learning during the SOP and JRD Tasks

**Figure 2:**
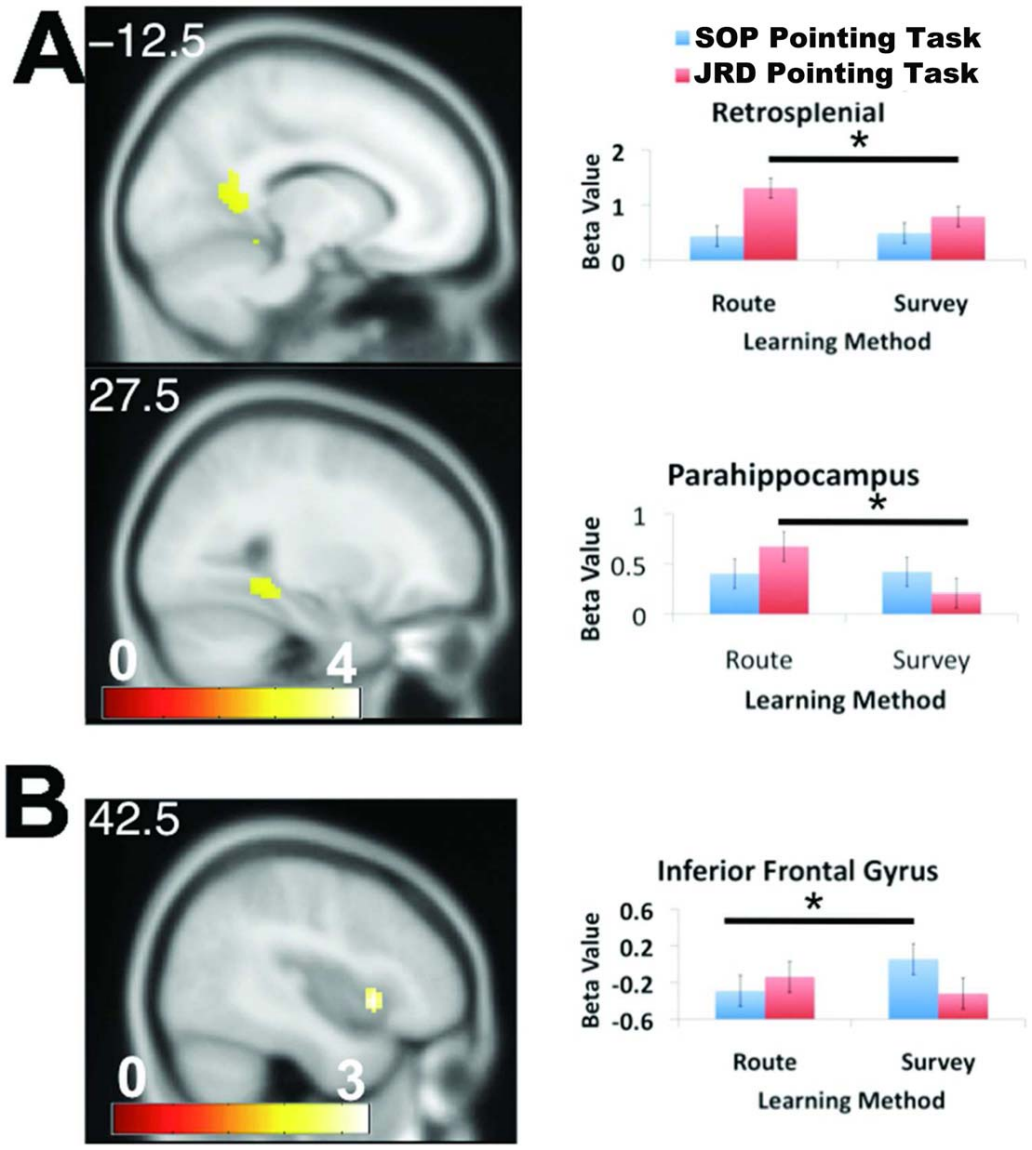
Brain regions showing differential activation following route ("route") vs. cartographic map learning ("survey") during the SOP vs. JRD tasks. A) Retrosplenial cortex ([14–52 12], z = 3.98) and parahippocampal cortex ([−26 −40 −12]) showed greater activation for the route > map contrast during the JRD but not SOP task. B) Inferior frontal gyrus [40 13 −2], z = 3.37) showed greater activation for the map> route contrast during the SOP but not JRD task.

To identify brain regions that differentially activated during the SOP and JRD pointing tasks following route vs. map learning, we identified clusters showing a condition by pointing task interaction effect. We then investigated these further with t-tests to understand the directions of these effects (see Methods). We found significant activations in retrosplenial, parahippocampal cortex, and inferior frontal gyrus (Figure 2, all tests p_FWE_<.05, see Methods). Subsequent analyses revealed that activations in these regions derived from different underlying effects. For the route > map contrast, retrosplenial cortex and parahippocampal cortex activated significantly during the JRD but not the SOP task (Figure 2A). Thus, both parahippocampal cortex and retrosplenial cortex activated to a greater extent during pointing to target landmarks in environments learned from a route perspective during the JRD but not SOP task. In contrast, for the map> route contrast, inferior frontal gyrus activated to a greater extent during the SOP but not the JRD task (Figure 2B). Thus, inferior frontal gyrus activated to a greater extent during pointing to target landmarks in environments learned from map a perspective during the SOP but not JRD task. No other interaction effects were significant.

**Figure 3:**
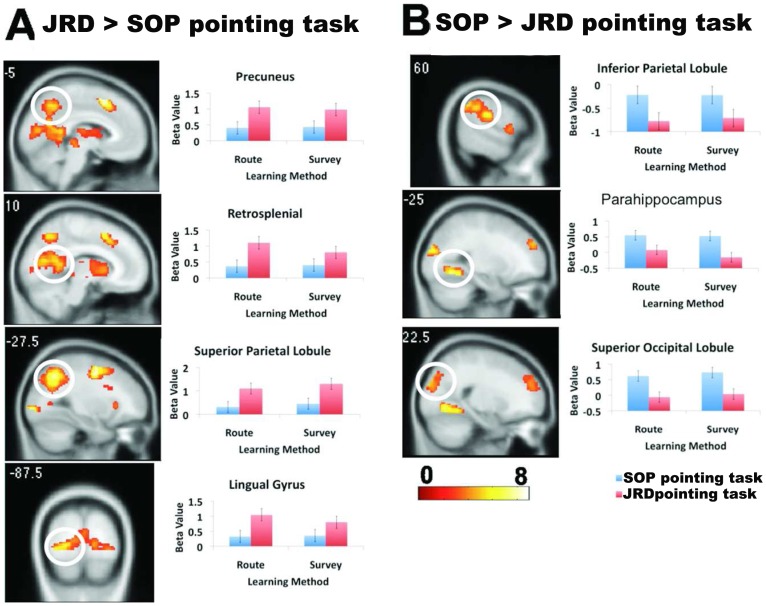
Brain regions showing greater activation in the A) JRD and b) SOP pointing tasks.

As explained in the Introduction, the SOP and JRD pointing tasks necessarily differed substantially in terms of their perceptual, orientation, and memory demands. This is because our primary aim was to understand how utilizing a representation formed during route learning might differ from that of map learning during these two tasks. Nonetheless, we thought it instructive to compare activations directly between the SOP and JRD task, in part because this might provide further insight into the differential effects we observed in the two tasks following route and map learning. A contrast of JRD > SOP blocks revealed clusters of significant activation in precuneus, retrosplenial cortex, superior parietal lobe, lingual gyrus, and other areas (Figure 3A and Table 2). These results suggest that these brain regions were comparatively more active during the JRD than the SOP task. A contrast of SOP> JRD task revealed clusters of significant activation in inferior parietal lobule, parahippocampal cortex, and superior occipital lobule (Figure 3B and Table 3). These results suggest that these brain areas were comparatively more active during the SOP than JRD task. No brain regions showed significant effects of route > map learning or vice versa, however, when we collapsed across JRD and SOP tasks.

**Table 2:** Spatial coordinates of clusters showing activation during the JRD > SOP comparison (p_FWE_ <0.05).

Region	Coordinate (x, y, z; in mm)		Voxel level (z-score)
	LH	RH	
Middle Frontal Gyrus	−28, −4, 48		9.39
	−3, 10, 52		7.77
	−40, 16, 28		7.55
		42, 30, 20	4.69
Retrosplenial	−13, −57, 12		7.39
Precuneus	−18, −62, 22		7.10
Superior Parietal Lobule	−28, −62, 42		7.08
Putamen	−18, 0, 15		6.07
		17, 13, 2	6.01
		20, 8, 15	5.54
Thalamus		24, −30, 10	3.89

RH, right hemisphere; LH, left hemisphere.

**Table 3:** Spatial coordinates of clusters showing activation during the SOP> JRD comparison (p_FWE_ <0.05).

Region	Coordinate (x, y, z; in mm)		Voxel level (z-score)
	LH	RH	
Parahippocampal Gyrus		30, −52, −8	7.47
	−26, −52, −10		5.67
Superior Occipital Gyrus		34, −84, 22	6.80
Middle Occipital Gyrus		40, −87, 10	5.57
		12, −97, 12	4.37
	−30, −90, 18		6.48
	−40, −87, 12		4.89
	−53, −72, 5		4.12
Fusiform Gyrus	−26, −62, −10		6.34
Cingulate Gyrus	−0, −20, 38		5.81
		4, −10, 40	4.80
		12, −27, 40	3.95
Inferior Parietal Lobule		60, −24, 25	5.73
		62, −40, 35	5.19
		67, −30, 35	4.90
	−56, −30, 25		4.98
	−63, −44, 35		4.03
	−58, −50, 40		2.95
Anterior Cingulate	−10, 48, −2		4.51
Superior Frontal Gyrus	−26, 50, 28		4.49
Middle Frontal Gyrus	−6, 46, 25		4.46
Insula	−46, −2, 0		4.50
	−40, 0, −10		3.87
		47, −4, 8	3.94
Claustrum	−38, −17, −2		4.46
Precentral Gyrus		57, 6, 8	4.08
Superior Temporal Gyrus		50, 3, −2	3.54
Inferior Frontal Gyrus		50, 36, −2	3.73
		47, 43, 8	3.18
		52, 26, 10	3.09

RH, right hemisphere; LH, left hemisphere.

## Discussion

In the current study, we employed a virtual reality paradigm in which participants learned spatial layouts by either actively navigating the environment (route learning) or studying a map based on an aerial view of the environment (map/survey learning). Participants then retrieved this information while undergoing fMRI with pointing tasks involving either egocentric orientation or referencing to external landmarks, suggested in previous behavioral work to be differentially affected based on route vs. map learning [10], [22]. In the SOP pointing task, participants navigated to a position at which they felt oriented prior to the beginning of each SOP block, ensuring that they were oriented, and then pointed to targets. In the JRD pointing task, participants viewed a blank screen and imagined themselves at one landmark, facing another, and pointed to a third landmark, ensuring they would be more likely to use a strategy involving referencing to external landmarks. Our task design thus allowed us to look at how route and cartographic map learning differentially affected the brain networks recruited during two substantially different spatial retrieval tasks studied extensively in past human spatial navigation behavioral studies. An important difference between the current study and previous behavioral studies mentioned is that in the current study, participants received more extensive training with spatial environments in both conditions to ensure a more balanced comparison in behavior between the SOP and JRD task during fMRI (see Methods). These manipulations thus allowed us to directly compare the effects of learning a spatial environment by directly navigating it vs. studying it from a map.

We found three brain areas that showed differential activations as a result of route and map learning: retrosplenial cortex, parahippocampal cortex, and inferior frontal gyrus. Parahippocampal and retrosplenial cortex showed higher activation for the route > map contrast during the JRD but not SOP task. Retrosplenial and parahippocampal cortex, though, showed overall different patterns of activation, with retrosplenial cortex showing greater activation during the JRD task and parahippocampal cortex showing greater activation during the SOP task. These data thus suggest that retrosplenial cortex played a more specific role in representation of landmark-referenced memory following route compared to map learning and overall during utilization of a more landmark-referenced representation compared to a more scene-dependent one. In contrast, while parahippocampal activation was greater during the JRD task following route compared to map learning, it generally showed greater activation during the SOP task compared to the JRD task, suggesting its more selective role in scene processing. A previous study by Epstein & Higgins showed that parahippocampal cortex played a specific role in processing the visuo-spatial structure of scenes while retrosplenial cortex played a specific role in placing scenes within the larger environment [23]. Our findings are consistent overall with these results, supporting the idea that parahippocampal cortex plays a more selective role in scene processing while retrosplenial cortex a more selective role in representation within the context of the larger spatial environment.

A principle difference one might expect for route compared to map learning is the need to integrate multiple viewpoints and trajectories during driving to form a holistic representation of the spatial layout. This idea predicts that even following extensive route learning, pointing relative to other landmarks should still necessitate some access to scene dependent information. One proposal of regarding the function of retrosplenial cortex in navigation is the conversion of scene and orientation dependent (egocentric) coordinates to landmark-referenced (allocentric) coordinates [24], [25], [26]. Consistent with this proposal, and our findings, patients with damage including the retrosplenial cortex show impairments in placing objects within a layout if they are rotated relative to the room (an external coordinate system) while they are unimpaired at placing these objects if the layout remains the same relative to their body position [27]. Thus, one possible conceptualization of the role of retrosplenial cortex in our experiment is that it plays a role in the active translation of egocentric-based, scene-dependent information to landmark-referenced allocentric coordinates. Similar to our findings for the retrosplenial cortex, we found greater parahippocampal cortex activation during the JRD task for the route > map contrast (but not for the SOP task). Because these findings are overall similar to what we found for retrosplenial cortex, the above ideas about the role of retrosplenial cortex in egocentric to allocentric conversion would also appear to hold for parahippocampal cortex. Parahippocampal and retrosplenial cortex differed, however, in their pattern of activation for the SOP vs. JRD task, with parahippocampal cortex showing greater activation during the SOP task and retrosplenial cortex showing greater activation during the JRD task. While this difference may relate to separate roles in scene-dependent vs. context dependent spatial memory, as suggested above, future work is required to better separate out their distinct roles in spatial memory.

Inferior frontal gyrus [activation centered at: 40 13 −2], in contrast, showed significant activation for the map> route contrast during the SOP but not JRD task. The SOP task required mental rotation of the spatial configuration relative to ones original position in the environment and one of our predictions was that map learning would increase the likelihood of representation of the environment as a distinct spatial configuration. Previous spatial memory studies have occasionally noted inferior frontal gyrus activation although this brain region is not often associated with spatial memory. In one such spatial memory study, Lambrey et al. (2011) had participants rotate and compare positions of objects on a table by either rotating the table relative to themselves or relative to other objects within the room [28]. In addition to several other areas of activation, the authors observed inferior frontal gyrus activation when participants had to rotate the table relative to the room compared to relative to oneself. Rotating the table could be considered analogous to rotating objects contained on a map while rotating relative to oneself might be analogous to rotating information from a route perspective. Because we found greater degrees of activation following map learning in the SOP task, one possibility is that the inferior frontal gyrus activation observed in both studies derived from rotating visual configurations using a geometrically-based strategy. Thus, one interpretation of our findings is that inferior frontal gyrus played a role in conversion of landmark-referenced coordinate information to one primarily dependent on orientation within the scene. Interestingly, phonological and sematic processing are often frequently associated with activation in inferior frontal gyrus (for a review, see: [29]), although one possible explanation of these findings may relate to a more general role for the IFG in response selection [30] and inhibition of competing responses [31]. Thus, an additional and valid interpretation of our findings, consistent with the response selection literature, is that performing the SOP task after map learning would necessitate inhibition of one’s current facing direction to rotate to a new facing direction in order to correctly point to the target. These demands may be reduced after route learning because these different facing directions have already been experienced directly. Future studies will be aimed at better characterized what differential processes are involved in converting a primarily landmark-referenced representation into a scene and orientation dependent one.

Our results, together with previous behavioral studies, provide support for the idea that neural-based representations formed following route and map learning rely on partially dissociable brain systems. Even after fairly extensive route learning, spatial representations may still depend, in part, on brain regions such as retrosplenial cortex involved in transforming scene-dependent representations to landmark-referenced ones. In contrast, cartographic map learning may depend on brain regions such as inferior frontal cortex that play roles in conversion from a geometrically-based coordinate system to a scene and orientation dependent one. Together, these results help to better define the differences between the neural basis of human spatial representation when formed from navigation vs. cartographic maps.

## Materials and Methods

### Ethics Statement

All participants gave written informed consent to participate in the study, which was approved by the institutional review board at the University of California, Davis.

### Participants

Sixteen participants (half female) were recruited from the general population in Davis, CA area. Participants were free of significant neurological deficits and had no history of psychiatric disorders, were right-handed, and had normal or corrected-to-normal vision.

### Experimental Stimuli

We used Panda3D software (Entertainment Technology Center, Carnegie Mellon University) to present two virtual cites. One of the cities was 100×130 virtual units and the other was 128×128 virtual units. To ensure little overlap between representations for the two different cities, different stores, landmarks, and geometry were employed for the two different layouts. Both were constructed to be as realistic as possible, consisting of a set of rectangular target stores (approximately 3.8×2.6×2.3 virtual units, length×wide×height), background buildings, trees, grass, trash cans, benches, walls, sky, and clouds. Target stores were arranged in such that participants could only view one store at a time while navigating (Figure 1C). The aerial view of the city was taken from a position 120 virtual units above the center of the virtual city. All the target stores and ground features were labeled so that participant could readily distinguish them from the aerial view (Figure 1A).

### Encoding via Route or Map Learning

During route learning, participants were instructed to learn the locations of stores in the virtual city, which they encountered by repeatedly driving to them (Figure 1C). A prompt in the upper left corner of the screen instructed which store to find. To ensure that participants were actually encoding the stores within the layout and not simply searching randomly until they found the store, after participants finished searching for each of the six target stores, they were asked to point to the direction of each store relative to their current position using their fingers. Participants were encouraged to search for the target store as quickly and directly as possible and pay attention to the location of each target store. This procedure repeated 5 times.

During map learning, participants were instructed to learn the locations of all stores from a map (Figure 1A) shown on the screen for 1 minute. The target stores were then removed from the map (Figure 1B) and participants were asked to locate each store on the map as accurately as possible. Participants repeated this procedure 5 times to ensure adequate learning of the environment before entering the scanner.

### SOP and JRD Pointing Tasks

fMRI occurred during both SOP and JRD pointing tasks and participants received practice on both pointing tasks prior to entering the scanner. For the SOP task, participants were placed in the virtual city (without the target stores) and were instructed to freely navigate the virtual city until they found a position where they were orientated (i.e., knew where they were). Participants navigated using a magnetic-compatible joystick (Current Design, Philadelphia, PA). The presence of other landmarks (e.g., buildings, roads, grass, etc) in the absence of target stores ensured that they were oriented without the possibility of learning additional information about target stores. We note that only a small subset of landmarks could be viewed at any given viewing angle (Figure 1), thus providing minimal additional information about the locations of targets other than providing a sense of orientation. At the beginning of each SOP pointing trial (Figure 1D upper row), the name of the target store was shown on the upper left corner of the screen (e.g., ‘Please point to the Camera Store’). Participants were instructed to think about the position of the target store based on their current position for 8 seconds (“cognitive part”). Immediately following, a virtual compass appeared at the bottom half of the screen and participants had 8 seconds to move the joystick to rotate the compass to the correct direction (“motor part”). Participants received explicit practice prior to imaging emphasizing that they should think of their answer during the cognitive part and provide their response during the motor part.

In the JRD task, participants were positioned with only the ground and sky in their field of view. We did this to avoid any stimuli which participants could use to orient themselves relative to the other landmarks in the city. At the beginning of each JRD pointing trial (Figure 1D lower row), instructions first appeared in the top half of the screen for 8 seconds (e.g., “Imagine you are standing at the Costume Shop, facing the Gym. Please point to the Camera Store”). These instructions required participants to imagine themselves in a comparatively novel position within the environment to better assay their knowledge of the configuration of stores (cognitive part). Then participants moved the joystick to rotate the compass to the correct direction (motor part). All subsequent analyses in the manuscript involve the cognitive part for both SOP and JRD pointing tasks to avoid possible contamination of activations with motor movements.

Immediately following a SOP or JRD pointing block, participants pointed to arrows facing either left or right as part of our baseline task [32]; the arrow pointing task continued for a minimum of 8 seconds. Each arrow appeared for 800 msecs and participants moved the joystick in the direction of the arrow.

Each trial of SOP or JRD pointing lasted 24 seconds, with the cognitive, motor parts, and baseline task lasting 8 seconds each. If participants finished pointing to the target store earlier during the second 8 seconds, the baseline task started earlier and lasted until 24 seconds total had elapsed for the trial. Following rotation of the joystick to the intended position, participants pressed a button on the joystick to confirm their selection. Trials on which directional selections were not confirmed within 8 seconds were excluded from all the analyses. Each block included 18 testing trials. The probability of each of the six selected stores appearing for any of the questions was fully randomized with the rule that one store could not appear twice in one trial and trials within the same block were not repeated. Four testing blocks were performed for each layout with half of them SOP pointing blocks and half JRD pointing blocks. The sequence of the SOP pointing and JRD pointing blocks was counterbalanced within and across participants. Before each block, participants were reminded of the layout to be tested. If the layout was encoded by route learning, a short video showing navigation through the layout with brief (3 second) stops at each store was shown to them. If the layout was encoded by map learning, a reminder map was showing to them for 1 minute. The design for testing the effects of route and map learning was completely within participant, with each participant learning one layout by driving and a different layout by map learning. The sequence of the two layouts, learning methods for the two layouts (route vs. map learning), and the testing sequence of two pointing tasks were all counterbalanced across participants.

### Behavioral Data Collection and Analysis

Pointing error was the offset of the actual pointing direction from the correct pointing direction. Configuration error in the SOP task was the standard deviation of the means per target object of the signed pointing errors [20]. This allowed us to account for situations in which a participant’s representation was rotated relative to the environment by a systematic offset (e.g., 180**°**) but otherwise correct.

### MRI Acquisition

Scanning was performed at the Imaging Research Center at the University of California, Davis on a 3T Siemens (Erlangen, Germany) Trio equipped with a thirty-two-channel head coil. Forty-three contiguous axial slices were acquired using a gradient-echo echo-planar T2*-sensitive sequence [repetition time (TR), 2000 ms; echo time (TE), 29 ms; voxel size, 2.5×2.5×2.5 mm; matrix size, 88×88×35]. Structural T1-weighted images for anatomical localization were acquired using a three-dimensional magnetization-prepared rapid-acquisition gradient echo pulse sequence [TR, 1900 ms; TE, 2.88 ms; inversion time (TI), 1100 ms; voxel size, 1×1×1 mm; matrix size, 256×256×208]. The first six volumes of each run were discarded to ensure stability of images. Foam padding was used to attenuate head motion. Visual stimuli were projected to a screen that could be seen from a mirror in the scanner and responses were collected using a magnetic compatible joystick.

### fMRI Data Analysis

Image processing and statistical analysis were performed using Statistical Parametric Mapping (SPM8, The Wellcome Department of Imaging Neuroscience, Institute of Neurology, London, UK). Functional images were motion-corrected and high-pass filtered to remove baseline drifts. After re-sampling the functional data at 1×1×1 mm and normalizing to the MNI template, the functional data were re-sliced to their original 2.5×2.5×2.5 mm resolution and smoothed with a 6 mm full-width at half-maximum (FWHM) isotropic Gaussian kernel. Data were first modeled for each participant individually using a general linear model (GLM); only trials on which participants responded were considered for further analysis. The first-level analysis provided parameter estimates for each condition and each participant against baseline. These parameter estimates were then entered into a repeated-measures, whole brain, random-effects 2×2 ANOVA (encoding method [route vs. map]×pointing task [egocentric vs. allocentric]) to determine activation patterns across the group.

### Correction for Multiple Comparisons

For the whole brain ANOVA analyses, we used a threshold of p_FWE_ <0.05, i.e., corrected for family-wise error (FWE). We did this by calculating the cluster size needed for a threshold of p_FWE_ <0.05 using Monte Carlo simulations [33] with 3dClustSim software based on an uncorrected, voxelwise p<0.005. These simulations showed that our threshold of p_FWE_<0.05 corresponded to p<0.005 with a voxel extent (k) of 38.

## References

[1] Wolbers T, Buchel C (2005) Dissociable retrosplenial and hippocampal contributions to successful formation of survey representations. J Neurosci 25: 3333–3340. 15800188 10.1523/JNEUROSCI.4705-04.2005 PMC6724902

[2] Tolman EC (1948) Cognitive Maps in Rats and Men. Psychol Rev 55: 189–208. 18870876 10.1037/h0061626

[3] O’Keefe J, Nadel L (1978) The Hippocampus as a Cognitive Map. Oxford: Clarendon Press.

[4] Klatzky R (1998) Allocentric and egocentric spatial representations: Definitions, Distinctions, and Interconnections. In: Freksa C, Habel C, Wender CF, editors. Spatial cognition: An interdisciplinary approach to representation and processing of spatial knowledge. Berlin: Springer-Verlag. 1–17.

[5] Thorndyke PW, Hayes-Roth B (1982) Differences in spatial knowledge acquired from maps and navigation. Cogn Psychol 14: 560–589. 7140211 10.1016/0010-0285(82)90019-6

[6] Moeser S (1988) Cognitive Mapping in a Complex Building. Environment and Behavior 20: 21–48.

[7] Richardson AE, Montello DR, Hegarty M (1999) Spatial knowledge acquisition from maps and from navigation in real and virtual environments. Mem Cognit 27: 741–750. 10.3758/bf03211566 10479831

[8] Hirtle SC, Hudson J (1991) Acquisition of spatial knowledge for routes. Journal of Environmental Psychology 11: 335–345.

[9] Sholl MJ (1999) Egocentric frames of reference used for the retrieval of survey knowledge learned by map and navigation. Spatial Cognition and Computation 1: 475–494.

[10] Taylor HA, Naylor SJ, Chechile NA (1999) Goal-specific influences on the representation of spatial perspective. Memory & cognition 27: 309–319. 10226440 10.3758/bf03211414

[11] Siegel AW, White SH (1975) The development of spatial representations of large-scale environments. In: Reese HW, editor. Advances in child development and behavior. New York: Academic. 10.1016/s0065-2407(08)60007-5 1101663

[12] McNamara TP, Hardy JK, Hirtle SC (1989) Subjective Hierarchies in Spatial Memory. Journal of Experimental Psychology-Learning Memory and Cognition 15: 211–227. 10.1037//0278-7393.15.2.211 2522511

[13] Shelton AL, Gabrieli JD (2002) Neural correlates of encoding space from route and survey perspectives. J Neurosci 22: 2711–2717. 11923436 10.1523/JNEUROSCI.22-07-02711.2002 PMC6758311

[14] Shelton AL, Pippitt HA (2007) Fixed versus dynamic orientations in environmental learning from ground-level and aerial perspectives. Psychol Res 71: 333–346. 16957953 10.1007/s00426-006-0088-9

[15] Latini-Corazzini L, Nesa MP, Ceccaldi M, Guedj E, Thinus-Blanc C, et al (2010) Route and survey processing of topographical memory during navigation. Psychological research 74: 545–559. 20174930 10.1007/s00426-010-0276-5

[16] Shelton AL, McNamara TP (2004) Orientation and perspective dependence in route and survey learning. J Exp Psychol Learn Mem Cogn 30: 158–170. 14736304 10.1037/0278-7393.30.1.158

[17] Mou W, McNamara TP, Valiquette CM, Rump B (2004) Allocentric and egocentric updating of spatial memories. J Exp Psychol Learn Mem Cogn 30: 142–157. 14736303 10.1037/0278-7393.30.1.142

[18] Rieser JJ (1989) Access to knowledge of spatial structure at novel points of observation. J Exp Psychol Learn Mem Cogn 15: 1157–1165. 2530309 10.1037//0278-7393.15.6.1157

[19] Holmes MC, Sholl MJ (2005) Allocentric coding of object-to-object relations in overlearned and novel environments. J Exp Psychol Learn Mem Cogn 31: 1069–1087. 16248751 10.1037/0278-7393.31.5.1069

[20] Waller D, Hodgson E (2006) Transient and enduring spatial representations under disorientation and self-rotation. J Exp Psychol Learn Mem Cogn 32: 867–882. 16822154 10.1037/0278-7393.32.4.867 PMC1501085

[21] Kelly JW, Avraamides MN, Loomis JM (2007) Sensorimotor alignment effects in the learning environment and in novel environments. Journal of experimental psychology Learning, memory, and cognition 33: 1092–1107. 10.1037/0278-7393.33.6.1092 17983315

[22] Rossano MJ, West SO, Robertson TJ, Wayne MC, Chase RB (1999) The acquisition of route and survey knowledge from computer models. Journal of Environmental Psychology 19: 101–115.

[23] Epstein RA, Higgins JS (2007) Differential parahippocampal and retrosplenial involvement in three types of visual scene recognition. Cereb Cortex 17: 1680–1693. 16997905 10.1093/cercor/bhl079

[24] McNaughton BL, Chen LL, Markus EJ (1991) “Dead Reckoning,” Landmark Learning, and the Sense of Direction: A Neurophysiological and Computational Hypothesis". Journal of Cognitive Neuroscience 3: 190–205. 23972093 10.1162/jocn.1991.3.2.190

[25] Byrne P, Becker S, Burgess N (2007) Remembering the past and imagining the future: a neural model of spatial memory and imagery. Psychological review 114: 340–375. 17500630 10.1037/0033-295X.114.2.340 PMC2678675

[26] Burgess N (2008) Spatial cognition and the brain. Ann N Y Acad Sci 1124: 77–97. 18400925 10.1196/annals.1440.002

[27] Hashimoto R, Tanaka Y, Nakano I (2010) Heading disorientation: a new test and a possible underlying mechanism. European neurology 63: 87–93. 20090342 10.1159/000276398

[28] Lambrey S, Doeller C, Berthoz A, Burgess N (2011) Imagining Being Somewhere Else: Neural Basis of Changing Perspective in Space. Cerebral cortex. 10.1093/cercor/bhr101 21625010

[29] Costafreda SG, Fu CH, Lee L, Everitt B, Brammer MJ, et al (2006) A systematic review and quantitative appraisal of fMRI studies of verbal fluency: role of the left inferior frontal gyrus. Human Brain Mapping 27: 799–810. 16511886 10.1002/hbm.20221 PMC6871344

[30] Thompson-Schill SL (2003) Neuroimaging studies of semantic memory: inferring “how” from “where”. Neuropsychologia 41: 280–292. 12457754 10.1016/s0028-3932(02)00161-6

[31] Aron AR, Robbins TW, Poldrack RA (2004) Inhibition and the right inferior frontal cortex. Trends in Cognitive Sciences 8: 170–177. 15050513 10.1016/j.tics.2004.02.010

[32] Stark CE, Squire LR (2001) When zero is not zero: the problem of ambiguous baseline conditions in fMRI. Proc Natl Acad Sci U S A 98: 12760–12766. 11592989 10.1073/pnas.221462998 PMC60127

[33] Forman SD, Cohen JD, Fitzgerald M, Eddy WF, Mintun MA, et al (1995) Improved assessment of significant activation in functional magnetic resonance imaging (fMRI): use of a cluster-size threshold. Magn Reson Med 33: 636–647. 7596267 10.1002/mrm.1910330508

